# Supplementary material for: Protective effects of the extracts of Barringtonia racemosa shoots against oxidative damage in HepG2 cells
Source: PeerJ. 2016 Jan 28;4:e1628. doi: 10.7717/peerj.1628 (PMC4734433; doi:10.7717/peerj.1628)
Supplement: Supplemental Information 1 [file peerj-04-1628-s001.pdf]

Data for Figure 2: The effects of gallic acid, BLE and BSE on cell viability (%) of HepG2 cells.

Treatment	GA (µg/ml)		BLE (µg/ml)		BSE (µg/ml)	
	Cell viability (%)	SEM	Cell viability (%)	SEM	Cell viability (%)	SEM
0	100.00	0.00	100.00	0.00	100.00	0.00
3.125	92.28	5.65	91.98	1.17	99.31	0.42
6.25	66.29	4.16	92.21	0.70	103.07	1.39
12.5	48.27	5.02	93.84	2.51	102.40	0.61
25	43.11	3.80	90.78	2.66	106.84	3.81
50	40.61	4.86	95.19	1.17	109.46	5.77
100	43.47	4.69	98.66	2.06	111.44	4.29
200	45.83	4.15	94.19	2.45	104.25	1.84
500	28.92	1.42	29.64	0.88	83.59	2.76

GA, gallic acid; BLE, leaf water extract of *B. racemosa*; BSE, stem water extract of *B. racemosa*; SEM, standard error of means.

Data for Figure 3: The effects of (A) gallic acid, (B) BLE and (C) BSE on antioxidant status (FRAP value, µM Fe^{2+}) of HepG2 cells.

Treatment	GA(µM)		BLE (µg/ml)		BSE (µg/ml)	
	FRAP (µM Fe^{2+})	SEM	FRAP (µM Fe^{2+})	SEM	FRAP (µM Fe^{2+})	SEM
Control	37.67	0.33	70.22	13.28	32.11	2.90
0.5	33.44	6.45	110.33	16.19	38.33	8.74
1	93.56	5.06	173.33	18.08	108.00	16.68
2	45.67	3.79	29.78	5.49	44.78	5.67
5	74.56	6.71	118.00	15.17	78.22	9.06
10	35.22	4.49	86.33	22.77	44.89	8.35
20	55.44	3.04	121.78	5.15	62.33	7.19

Control, untreated cells; GA, gallic acid; BLE, leaf water extract of *B. racemosa*; BSE, stem water extract of *B. racemosa*; SEM, standard error of means.

Data for Figure 4: The cytoprotective effects of (A) gallic acid, (B) BLE and (C) BSE on HepG2 cells following H_2O_2-induced oxidative damage.

Treatment	GA (µM)		BLE (µg/ml)		BSE (µg/ml)	
	Cell viability (%)	SEM	Cell viability (%)	SEM	Cell viability (%)	SEM
Control	100.00	2.26	100.00	1.39	100.00	1.22
H_2O_2	31.97	1.43	27.57	2.48	26.27	1.70
0.5 + H_2O_2	32.90	1.90	26.21	2.42	18.89	1.64
1 + H_2O_2	51.59	4.08	38.71	1.55	33.22	1.69
2 + H_2O_2	24.78	0.25	19.76	2.94	15.51	0.93
5 + H_2O_2	42.87	2.05	26.39	2.63	20.67	1.21
10 + H_2O_2	32.93	0.97	18.08	1.78	19.74	1.72
20 + H_2O_2	35.74	0.59	26.98	2.82	19.91	0.82

Control, untreated cells; H_2O_2: positive control; GA, gallic acid; BLE, leaf water extract of *B. racemosa*; BSE, stem water extract of *B. racemosa*; SEM, standard error of means.

Data for Figure 5: The effects of (A) gallic acid, (B) BLE and (C) BSE on ROS production of HepG2 cells following H_2O_2-induced oxidative damage.

Treatment	GA (µM)		BLE (µg/ml)		BSE (µg/ml)	
	RFU	SEM	RFU	SEM	RFU	SEM
Control	67.33	0.33	69.43	0.96	68.75	0.68
H_2O_2	73.84	0.36	86.25	3.32	79.59	1.22
0.5 + H_2O_2	75.25	0.44	66.94	0.16	66.09	0.77
1 + H_2O_2	72.03	0.97	66.15	0.43	63.03	0.40
2 + H_2O_2	68.68	0.16	66.45	1.12	60.75	0.32
5 + H_2O_2	69.70	1.64	62.69	0.23	62.55	0.36
10 + H_2O_2	69.36	1.21	65.02	1.48	61.64	0.19
20 + H_2O_2	66.50	1.60	69.71	0.45	66.10	0.08

Control, untreated cells; $H_2O_{2,}$ positive control; GA, gallic acid; BLE, leaf water extract of *B. racemosa*; BSE, stem water extract of *B. racemosa*; SEM, standard error of means. RFU, relative fluorescence unit.

Data for Figure 6: The effects of (A) gallic acid, (B) BLE and (C) BSE on lipid peroxidation of HepG2 cells following H_2O_2-induced oxidative damage.

Treatment	GA (µM)		BLE (µg/ml)		BSE (µg/ml)	
	MDA (nmol/g protein)	SEM	MDA (nmol/g protein)	SEM	MDA (nmol/g protein)	SEM
Control	15.39	3.51	15.39	3.51	15.39	3.51
H_2O_2	51.86	10.80	51.86	10.80	51.86	10.80
0.5 + H_2O_2	24.02	4.27	18.58	8.77	39.24	5.11
1 + H_2O_2	16.20	3.61	36.67	9.57	32.11	8.79
2 + H_2O_2	33.15	6.15	17.65	4.39	23.59	7.15
5 + H_2O_2	17.70	7.05	22.17	8.35	22.56	6.76
10 + H_2O_2	30.30	8.83	34.27	9.84	19.29	5.95
20 + H_2O_2	27.71	6.97	8.05	2.74	20.80	1.26

Control, untreated cells; H_2O_2, positive control; GA, gallic acid; BLE, leaf water extract of *B. racemosa*; BSE, stem water extract of *B. racemosa*; SEM, standard error of means; MDA, malondialdehyde.

Figure 7 The effects of gallic acid, BLE and BSE on activities of SOD (A-C) and CAT (D-F) in HepG2 cells following H_2O_2-induced oxidative damage.

Superoxide dismutase:

Treatment	GA (µM)		BLE (µg/ml)		BSE (µg/ml)	
	SOD (U/mg protein)	SEM	SOD (U/mg protein)	SEM	SOD (U/mg protein)	SEM
Control	7.90	1.01	7.90	1.01	7.90	1.01
H_2O_2	20.74	2.83	20.74	2.83	20.74	2.83
0.5 + H_2O_2	11.62	0.36	12.85	2.35	8.16	1.35
1 + H_2O_2	13.02	1.92	4.40	1.92	6.93	2.07
2 + H_2O_2	16.16	1.78	13.51	3.91	6.02	0.59
5 + H_2O_2	10.26	0.49	21.80	4.65	17.36	1.85
10 + H_2O_2	11.08	2.49	17.44	5.04	6.59	1.60
20 + H_2O_2	9.67	2.93	13.11	2.67	8.22	1.01

Control, untreated cells; H_2O_2: positive control; GA, gallic acid; BLE, leaf water extract of *B. racemosa*; BSE, stem water extract of *B. racemosa*; SEM, standard error of means.

Catalase:

Treatment	GA (µM) CAT (U/mg protein)	SEM	BLE (µg/ml) CAT (U/mg protein)	SEM	BSE (µg/ml) CAT (U/mg protein)	SEM
Control	117.47	11.19	117.47	11.19	117.47	11.19
H_2O_2	183.25	12.28	183.25	12.28	183.25	12.28
0.5 + H_2O_2	131.22	18.18	224.46	28.41	189.34	16.44
1 + H_2O_2	155.11	1.47	162.00	3.96	148.46	18.27
2 + H_2O_2	172.36	7.62	134.08	12.45	126.13	15.06
5 + H_2O_2	144.93	14.19	184.33	6.97	248.14	6.66
10 + H_2O_2	121.85	5.79	208.30	4.52	175.69	18.29
20 + H_2O_2	135.85	10.53	139.52	20.62	167.58	25.10

Control, untreated cells; H_2O_2: positive control; GA, gallic acid; BLE, leaf water extract of *B. racemosa*; BSE, stem water extract of *B. racemosa*; SEM, standard error of means.
